# This is our lane: talking with patients about racism

**DOI:** 10.1186/s40695-021-00066-3

**Published:** 2021-08-28

**Authors:** Michelle S. Diop, Christy N. Taylor, Sascha N. Murillo, Jessica A. Zeidman, Aisha K. James, Sherri-Ann M. Burnett-Bowie

**Affiliations:** 1 Department of Medicine, Massachusetts General Hospital and Harvard Medical School, Boston, MA USA; 2 Division of General Internal Medicine, Department of Medicine, Massachusetts General Hospital and Harvard Medical School, Boston, MA USA; 3 Department of Pediatrics, Massachusetts General Hospital and Harvard Medical School, Boston, MA USA; 4 Endocrine Division, Department of Medicine, Massachusetts General Hospital and Harvard Medical School, 50 Blossom Street, Thier 1051, Boston, MA 02114-2696 USA

**Keywords:** Discrimination, Racism, Bias, Trauma, Structural competency, Asian, African American, Black, Latinx, BIPOC

## Abstract

Racism has significantly impacted communities of color for centuries. The year 2020 is a reminder that racism is an ongoing public health crisis. Healthcare institutions have an important role in dismantling racism because of their ability to implement innovative solutions that advance diversity, address social determinants of health, and promote health equity. Healthcare professionals have the unique opportunity to support patients by discussing patients’ experiences of bias and racism. Asking about discrimination, however, can be difficult because of the sensitive nature of the topic and lack of appropriate education. This review highlights the importance of addressing patients’ experiences of racism, utilizing the frameworks of trauma-informed care, structural competency, provider bias, and intersectionality. Furthermore, this review provides ways to engage in meaningful dialogue around discrimination and includes important patient-centric resources.

## Introduction

2020 was marked by local and global events that created a national reckoning with the impact of racism on the health and well-being of people of color in the United States (US). The COVID-19 pandemic has inflicted excess harm on Asian, Black, Latinx, and Indigenous communities. Such disparities can be attributed to a long history of US policies and practices that resulted in members of these communities living in multigenerational households, having greater reliance on public transportation, and having more front-line occupations with higher risk of COVID-19 exposure [1, 2]. Disturbingly, the COVID-19 pandemic has been associated with a significant rise in anti-Asian racism, which has culminated in almost 3800 attacks on people of Asian descent [3]. The killings of Mr. George Floyd, Mr. Ahmaud Arbery, and Ms. Breonna Taylor in 2020 sparked a conversation on how structural racism [4], bias and discriminatory policies and practices that result in inequitable distribution of resources and opportunities, and interpersonal racism [4],bias and discriminatory behaviors and attitudes that occur between individuals, result in disproportionate harm to Black communities in the US.

The American Public Health Association (APHA) considers racism a public health crisis [5]. Indeed, many healthcare organizations and professional medical societies have reaffirmed their commitment to addressing racism and promoting health equity [6–8]. Healthcare organizations have approached this commitment in a variety of ways, from seeking to address racial biases and inequities within their institutions, to tackling structural racism by addressing social determinants of health in the community [9–11]. In particular, increased attention is being given to discussing the impact of racism and other forms of discrimination directly with patients [7, 12, 13]. However, outside of psychotherapy and pediatric literature, there is a dearth of research and resources in the medical field that is dedicated to assisting clinicians with having these conversations [7, 12, 14–16].

In this review, we aim to demonstrate the importance of acknowledging and addressing experiences of racism directly with patients. We draw on various theoretical frameworks to illustrate possible approaches for integrating such discussions in the clinical encounter. While the focus is on discussing experiences of racism with patients, these approaches can also be applied to discussions with patients about experiences of discrimination related to sex, gender identity, sexual orientation, disability, immigration status, etc.

## Why inquire about racism?

Camara Phyllis Jones, MD, MPH, former president of the APHA, stated that “racism is a system of structuring opportunity and assigning value based on the social interpretation of how one looks (race),” that unfairly disadvantages some individuals and communities and unfairly advantages others [17]. Racism operates at various levels in society, and it is important to understand the many ways that racism manifests. Racism can be internalized and manifest as lower scores on standardized testing when students are reminded of their racial/ethnic group (stereotype threat) [4, 18]. Additionally, racism can operate at the interpersonal level whereby racial bias occurs between individuals; at the institutional level (schools, workplaces, etc.) where discriminatory policies can routinely produce racial inequities; and at the structural level, which accounts for the cumulative historical and societal factors that have led to systematic disadvantage of particular racial groups [4].

Racism and discrimination are fundamental determinants of health and illness [19–26]. Discrimination is a form of stress that can affect both physical and mental well-being [27, 28]. This stress contributes to wear and tear on the body, or allostatic load, which can result in poor health, including impaired cardiovascular health, metabolic disarray, a weakened immune system, low self-esteem, depression, and anxiety [29–33]. Moreover, there is the additional impact of discrimination that occurs within healthcare settings. In a recent report, 1 in 5 Black adults reported having experienced race-based discrimination in healthcare settings in the last year, and participants were overall less likely to express trust in their doctors or the healthcare system [34]. Mistrust and discriminatory experiences discourage patients from seeking care, limit their ability to follow recommendations, and hinder the development of strong patient-physician relationships [33–35]. Mistrust of the healthcare system is deeply rooted in the historical exploitation of Black people by the scientific and medical community. Notable examples include the use of Mrs. Henrietta Lacks’ cells for medical and scientific advancement and the Tuskegee Syphilis Study that examined the natural course of syphilis in Black men for decades, even after the availability of curative treatment [36, 37]. Providers must be aware of the historical and current-day factors that contribute to patients’ mistrust of the healthcare system; empowered with this knowledge, providers can then create safe spaces within the clinical encounter that build trust and engagement.

## Approaches to discussing racism with patients: trauma-informed care as a framework

Beyond mistrust of the healthcare system, racism and discrimination have led to significant trauma [16, 38]. A systematic review investigating racism and trauma found a moderate to strong positive association between experiences of racial discrimination and trauma symptoms [38]. Race-based trauma includes cumulative experiences of racism and discrimination over one’s lifetime as well as intergenerational trauma [16, 38].

Trauma‐informed care provides an approach to discussing trauma from racism with patients. Trauma-informed care acknowledges and addresses the intersection and cumulative effects of interpersonal and structural forms of violence on people’s lives and health [39]. Trauma-informed care has been used in the setting of intimate partner or gender-based violence; and has been integrated into social services, behavioral health, and medical settings. Race-based traumatic stress injury is a consideration in mental healthcare [16]. Racism and discrimination can take many forms, such as racism in the form of systematic exclusion (e.g., voter suppression), interpersonal racism (e.g., microaggressions), or discrimination (e.g., denial of promotion at work) [16]. Racism occurs in a sociocultural context and the emotional impact of racism should not be attributed to an individual’s failings [16].

The Substance Abuse and Mental Health Service Administration (SAMHSA) provides guidance for implementing trauma-informed approaches in behavioral health services delivery, which can be extrapolated to other healthcare settings [40]. SAMHSA emphasizes the importance of developing a shared understanding among care providers of the impact of trauma on patients, implementing trauma screening and assessment, developing policies to respond to trauma at the individual and institutional level, and resisting re-traumatization [40]. SAMHSA further delineates six key principles to a trauma-informed approach: creating a culture and environment of safety, maintaining transparency and trust, providing peer support and connection around trauma, fostering collaboration between staff and patients, empowering patients and staff, and promoting an understanding of trauma in relation to structural and historical factors [40].

Discussing discrimination can lead to re-traumatization and patients may struggle with disclosing past experiences due to feelings of shame or concerns about stigma [41]. Thus, it is important to use trauma-informed care as a universal precaution so that care can be delivered without causing harm. Moreover, providers do not need to know the details of the trauma to exert a positive influence [41, 42]. Continuing education and training on discussing discrimination should improve providers’ competence and increase screening for discrimination [43].

## Microaggressions and healthcare

Microaggressions are brief and subtle verbal or non-verbal negative messages directed towards members of minority identity groups [44–46]. In addition to experiencing microaggressions in the wider society, patients may experience microaggressions in the healthcare setting [14, 46–53]. Similar to members of the wider community, healthcare providers have unconscious or implicit bias [54, 55], which can manifest as microaggressions [46, 53]. Additionally, provider-held unconscious bias can negatively impact care delivery and patients’ health outcomes, despite providers’ best intentions [54, 56–58]. Thus, it is imperative that healthcare providers focus on reducing their unconscious bias and learn about microaggressions, which can be done through self-directed learning or curricula that many hospitals are implementing. Healthcare providers should utilize frameworks and language that are inclusive and respect affirming [59], as well as appreciate that certain phrases or questions may be offensive or stigmatizing [46, 60, 61]. Moreover, continued emphasis on understanding the lived experiences of patients and exercising empathy are critical to building a harmonious relationship with the diverse populations whom we serve [55].

## Considerations for discussing racism with patients: structural competency

A key component of implementing a trauma-informed approach to effectively screening and responding to patients’ experiences of discrimination involves educating providers on the structural and historical forces that fuel racism. Experiences of racism and other forms of discrimination are the result of historical and current political and institutional factors. Understanding these structural drivers of racism is important, as research suggests that healthcare providers are more likely to attribute health inequities to individual characteristics as opposed to structural barriers [62, 63]. Providers should therefore have a shared understanding of how structural racism impacts patients before asking patients about those experiences. Structural competency is a framework that can be used to educate providers on how to understand and address the effects of discrimination on health and well-being [64–66].

Structural competency promotes an understanding of how forces, such as racism, impact health and exacerbate inequities (e.g., residential segregation affecting access to healthcare services), and how those forces can also shape the clinical encounter (e.g., implicit biases that shape provider perceptions about patients on the basis of race). Structural competency also advocates for clinician engagement in identifying structural solutions, for example, by having clinicians address food insecurity for patients with diabetes struggling to adhere to dietary recommendations. Structural competency curricula have been employed at various levels of clinician training and across a variety of disciplines within healthcare [67–70]. The principles of structural competency can be used to develop trainings and curricula for providers as part of implementing racism and discrimination screening tools or practices. Such a curricula could include education on the history of racism in medical research, the social construct of race within medicine, and the various policies that led to persistent racial inequities in housing, food access, and wealth. Empowered with this knowledge, clinicians should feel better prepared to discuss racism with patients.

## Considerations for discussing racism with patients: provider-held bias

In addition to cultivating a comprehensive understanding of the structural components of racism, clinicians should develop an awareness of provider-held biases that may affect their clinical interactions and screening practices. Integrating discussions about experiences of racism into the clinical encounter requires that providers acknowledge and address individually-held biases that could impact such conversations. Implicit bias refers to “learned stereotypes and prejudices that are automatically and unconsciously exercised” [55, 71, 72]. Notably, implicit bias involves both favorable and unfavorable attitudes about different groups and can lead to preferential treatment of certain groups.

Implicit bias has been associated with diagnosis disparities, treatment disparities, and lower quality of care [54, 56, 73]. Clinicians with more bias against Black people have been consistently evaluated as providing less patient-centered care by their Black patients as compared to clinicians with little or no such implicit bias [74]. Several systematic reviews have examined provider-held bias and its impact on healthcare [54, 56, 73, 75–79]. Notably provider-held implicit racial bias has been associated with racial disparities in pain management and maternal healthcare [54, 56, 73].

To address clinician-held bias and create a safe space for all patients, both systems and individuals need to adopt new practices. The implicit association test (IAT) is a tool to assist providers in recognizing and acknowledging their own biases as a means to understanding how these biases can lead to poor health outcomes for patients [71, 80]. To mitigate against bias, there must be both individual and institutional action. Individuals should continuously reflect upon their attitudes and actions related to bias. For example, clinicians should feel empowered to intervene if they witness overtly or covertly biased exchanges or if they identify a racist policy or process. At the institutional level, organizations should actively reflect on their engagement with internal and external communities, delivery of education, approach to clinical care, conduct of research, and approach to leadership development from an equity or anti-racism lens. Additionally, to facilitate the needed culture change, institutions must equip providers with structural competency education so that providers understand how racism is impacting their patients’ experiences and how racism (overt or covert) is impacting the delivery of care and other institutional missions.

## Considerations for discussing racism with patients: intersectionality

When asking about a patient’s experience of racial discrimination, it is important to recognize that individuals hold various identities that interact with each other and that impact the experience of discrimination. Women of color, who are in the mid-life, may have experienced the combined negative impacts of racism, sexism (discrimination on the basis of sex or gender), and ageism (discrimination on the basis of age), a framework called, intersectionality. Kimberly Crenshaw shared that, “Black women can experience discrimination in ways that are both similar and different from those experienced by White women and Black men” [81]. Transwomen of color may experience particularly high levels of discrimination and adverse health outcomes [60, 62]. Discrimination experienced by women of color in midlife is associated with increased risk of hypertension and metabolic syndrome, higher levels of C-reactive protein, a nonspecific marker of inflammation, as well as reduced breast and cervical cancer screening [82–84]. Given the interconnectedness of social identities, discrimination, and health outcomes, it is particularly important for providers to create space during clinical encounters for patients to share their experiences. In so doing, providers will better understand the social factors impacting patients’ health.

## If you screen what do you say?

Discussing discrimination, and racism specifically, is challenging and especially difficult if providers feel unequipped to do so and constrained by time. Similar to other topics, starting with open-ended questions (Table 1), e.g., “How are you doing during this challenging time” allows for the topic to be introduced in a general way. Follow-up questions or statements include: “How are you feeling about what’s going on in the world/our country right now as it relates to racism;” [85] or “The experience of racism is a major contributor to stress. As your physician, I would like to create a safe space for you to discuss key life experiences that contribute to your mental and physical health. Would you be comfortable talking with me about some of your experiences?” If permission is not given let your patient know that you are there for them in the future. Additional questions include those from the Everyday Discrimination Scale (“Do you feel like you are treated with less respect than other people? Do you feel like you are treated unfairly at restaurants or stores? Do you feel discriminated against in other ways?”[86]) and those that reflect discrimination related to being a member of different minority groups (“If you have experienced discrimination because of a combination of factors, for example, your gender and your age, we can talk about that too”) [87].

**Table 1:** Examples of screening questions for discrimination

How are you doing during this challenging time?
How are you feeling about what’s going on in the world/our country right now as it relates to racism [85]?
As your physician, I would like to create a safe space for you to discuss key life experiences that contribute to your mental and physical health. Would you be comfortable talking with me about some of your experiences?^a^
Many of my patients experience racism in health care. Are there any experiences that you would like to share with me [15]?
Are there important life events that you’ve experienced that have or are currently affecting your health [15]?
Do you feel like you are treated with less respect than other people [86]?
Do you feel like you are treated unfairly at restaurants or stores [86]?
Do you feel discriminated against in other ways [86]?
How has this affected your everyday life?
How have you dealt with this level of stress?
If you have experienced discrimination because of a combination of factors, for example, your gender and your age, we can talk about that too [87].
Is there anything that you would like to tell a future physician, nurse, or other healthcare provider?

^a^If permission is not given, let your patient know that you are there for them in the future

As healthcare providers, we must also recognize the power differential that exists between providers and patients. This power dynamic, may at times, prevent discussion of sensitive topics such as discrimination. This is likely exacerbated by ethnic or racial discordance between providers and patients. Given the power differential, it is important to assuage patients’ concerns through emphasizing allyship and to appreciate that it might take time for a patient to disclose their experiences with discrimination. Phrases such as those mentioned in Table 1 provide a framework for broaching these discussions in a gentle manner.

The Southern Jamaica Plain Health Center toolkit for addressing racial justice in the clinical setting includes suggested language for discussing race and racism with patients [15]. When asking about experiences in the healthcare system, providers could say “Many of my patients experience racism in healthcare. Are there any experiences that you would like to share with me?” With regards to life experiences, a clinician could ask: “Are there important life events that you’ve experienced that have or are currently affecting your health?” This toolkit includes the following key approaches: thanking the individual for sharing their story; validating the experience; and adopting a position that acknowledges the injustice, trauma, and pain, and which also affirms the patient’s dignity and strengths. Finally, providers should commit to partnering with patients to address their needs and incorporate their concerns in a shared care plan (Table 1) [15].

## If you screen what should you do next?

When a patient shares a discriminatory experience, the provider must actively listen and validate the patient’s experience. It is important to acknowledge the impact of the experience on the patient and avoid attempts to explain or defend the perceived act of discrimination. Providers should offer help through continued conversation and additional support if needed. Patients may experience mental health sequelae from racism or discrimination and benefit from referral to psychiatry and behavioral health services (Table 2). Patients who experience discrimination in the healthcare setting should be connected to local patient advocacy programs or national organizations, if local services are unavailable (Table 3). Those who experience discrimination related to employment, housing, education, or the criminal justice system may need to be directed to civil rights legal aid. Healthcare institutions and individual clinicians should commit to developing pathways to support patients impacted by racism. For example, medical-legal partnerships are one model of addressing social and structural determinants of health by integrating legal services within the healthcare setting.

**Table 2:** Suggested next steps after a patient discloses experiences of discrimination

Acknowledge the impact on the patient; let the patient know that they are not at fault and did not deserve it.
Avoid attempts to explain or defend the perceived act of discrimination.
Offer help through continued conversation.
Respect autonomy and the patient's right to make decisions about what to do and when.
Consider referral to psychiatry and behavioral health services if patient is amenable.
Consider connecting patient to a local patient advocacy program or civil rights legal aid.

**Table 3:** Resources to address discrimination in healthcare

NAACP local branches (https://www.naacp.org/find-local-unit/)
Patient advocacy or relations department within hospital
US Department of Health and Human Services complaint portal (https://www.hhs.gov/civil-rights/filing-a-complaint/complaint-process/index.html?language=en)
US Department of Justice Civil Rights Division reporting (https://civilrights.justice.gov/#report-a-violation)
Joint Commission Office of Quality and Patient Safety submission (https://www.jointcommission.org/resources/news-and-multimedia/the-joint-commission-stands-for-racial-justice-and-equity/)

## Impact on providers of color

While it is important for providers to create a safe space for patients to share their experience of racism, providers should also appreciate how such discussions affect their own well-being. Discussing patients’ experiences of discrimination or racism can be difficult for providers of color because it may remind them of their own experiences of discrimination or racism. Conversely, having these conversations may empower providers of color through their creation of a safe space for patients with whom they share racial or ethnic identity.

## Impact on providers who self-identify as White

Discussing racism may raise other challenges. Inquiring about experiences of racism requires that White providers confront race-based discrimination that they may be unaware of or that they may have been socialized to not believe in. Furthermore, conversations about a patient’s experience of discrimination may lead a White provider to appreciate the privilege that they experience because of race, which may have previously been invisible to them. The process of screening a patient about discrimination may thus cause a White provider to experience discomfort that can act as a barrier to continued screening. Hearing about patients’ experiences of discrimination may also provoke other emotions, such as sadness and anger, among White providers. Despite not experiencing this trauma personally, when White providers listen to patients’ experiences of racial trauma, it can shift their world view, a phenomenon called vicarious trauma [88].

## Impact on all providers

Vicarious trauma can impact healthcare providers of all racial/ethnic backgrounds. Clinicians should appreciate that the continuous exposure to vicarious trauma can lead to compassion fatigue and possibly burnout within themselves [88]. Balancing emotions through meditation, prioritizing social connections with family or loved ones, and exercise are a few examples of ways to mitigate vicarious trauma [89]. Finally, using the lens of trauma-informed care as aforementioned is critical in advancing the provider-patient relationship while preventing re-traumatization.

## Conclusions

The events of 2020 highlight the ways in which racism affects the daily realities of communities of color. It is of paramount importance that healthcare providers equip themselves with an understanding of how racism impacts patients, provide space for patients to discuss their experiences, and connect patients with resources and support. Trauma-informed care frameworks may assist providers and healthcare institutions in having these conversations with patients. Additionally, structural competency curricula and implicit bias training may serve as important resources to provide clinicians with social, historical, and political context and personal awareness prior to interacting with patients. Importantly, toolkits on eliciting patients’ experiences of racism exist. Finally, patients’ disclosures about racism must be affirmed and providers should know the behavioral health and legal resources that are available for patients, who are navigating experiences of racism. Discussing racism with our patients—this is our lane.

## Data Availability

Not applicable.

## Abbreviations

US: United States
APHA: The American Public Health Association
SAMHSA: Substance Abuse and Mental Health Service Administration
IAT: Implicit association test
